# Comparative Effectiveness of Pessary Placement, Cervical Cerclage, or Expectant Management in Preventing Preterm Delivery in Twin Pregnancies

**DOI:** 10.3390/jpm16020104

**Published:** 2026-02-10

**Authors:** Christina Pagkaki, Nektaria Kritsotaki, Anastasia Bothou, Vasiliki Kourti, Georgios Tsatsaris, Barbara Niesigk, Efthymios Oikonomou, Nikolaos Machairiotis, Nikolaos Tsikouras, Spyridon Michalopoulos, Anastasia Grapsa, Angeliki Gerede, Nikoletta Koutlaki, Alexander Tobias Teichmann, Panagiotis Tsikouras

**Affiliations:** 1 Department of Obstetrics and Gynecology, Democritus University of Thrace, 68100 Alexandroupoli, Greece; christinapagkaki@gmail.com (C.P.); nick.tsik.2001@gmail.com (N.T.); agerede@otenet.gr (A.G.);; 2 Department of Midwifery, School of Health Sciences, University of West Attica (UNIWA), 12243 Egaleo, Greece; 3 Department of Obstetrics and Gynecology, Clinicum Freudenstadt, 72250 Freudenstadt, Germany; 4 Department of Obstetrics and Gynecology, Clinicum Aschaffenburg, Teaching Hospital University of Würzburg, 63739 Aschaffenburg, Germany; 5 Third Department of Obstetrics and Gynecology, University General Hospital “ATTIKON”, Medical School, National and Kapodistrian University of Athens, 12462 Athens, Greece; 6 Department of Microbiology, University Hospital Alexandroupolis, 68100 Alexandroupolis, Greece; anastasiagrapsa@yahoo.gr

**Keywords:** personalized medicine, Arabin pessary, cervical cerclage, twin pregnancies, preterm birth

## Abstract

**Objective:** The objective of this study was to evaluate the association between cervical management strategies, specifically pessary placement, cervical cerclage, or expectant management, and gestational age at delivery in twin pregnancies and to assess the prognostic value of cervical characteristics for early preterm birth (<33 weeks). **Methods**: We conducted a retrospective cohort study including 120 twin pregnancies managed at a tertiary referral center between 2019 and 2024. Pregnancies with positive vaginal or cervical microbiological cultures or abnormal cervical cytology were excluded. The management strategy was selected based on cervical characteristics and clinical judgment. Gestational age at delivery was compared across intervention groups using descriptive statistics, kernel density plots, boxplots, and Kaplan–Meier survival analysis. Multivariable Cox proportional hazards regression was performed to estimate adjusted hazard ratios (HRs) for early delivery, including intervention type and cervical parameters (length, diameter, and funneling). **Results**: Overall, 26 of 120 pregnancies (21.6%) resulted in delivery before 33 weeks. Pessary placement was associated with longer gestational duration compared with cerclage or expectant management. Kaplan–Meier analysis demonstrated a clear separation of survival curves by intervention group, with the pessary group maintaining pregnancy to later gestational ages (log-rank *p* < 0.001). In multivariable Cox regression analysis, pessary use was associated with a significantly lower hazard of early delivery compared with cerclage (HR = 0.088, 95% CI: 0.035–0.220; *p* < 0.001). Expectant management showed a trend toward an increased risk of early delivery (HR = 2.44; *p* = 0.067). Cervical length and diameter were not independently associated with early delivery after adjustment for intervention type. Funneling was associated with a lower hazard of early delivery, a finding that warrants cautious interpretation. **Conclusions**: In this retrospective cohort of twin pregnancies without microbiological evidence of infection, pessary placement was associated with prolonged gestation and a lower hazard of early preterm delivery compared with cerclage or expectant management. These findings support a personalized, risk-adapted approach to cervical intervention selection in twin pregnancies. Prospective, randomized studies incorporating etiologic stratification are needed to confirm these associations and guide clinical practice.

## 1. Introduction

Preterm birth (PTB) remains the leading cause of neonatal morbidity and mortality and appears with increased incidence in multiple gestations, especially in twin pregnancies. An estimated 10% of twin pregnancies are delivered preterm, before 32 weeks of gestation [1]. However, applying proper preventive measures to this group remains a major challenge, largely due to heterogeneity in individual risk profiles and cervical characteristics.

Cervical cerclage has long been used in singleton pregnancies with a previous history of preterm delivery (PTB) or a short cervix. In twin pregnancies with asymptomatic second-trimester dilation, diagnosed by clinical examination, a randomized controlled trial showed that cerclage significantly lowered PTB rates (<34 weeks: 70% vs. 100%, <28 weeks: 41% vs. 84%) and decreased perinatal mortality (17.6% vs. 77%) compared with conservative management using indomethacin and antibiotics [2]. Systematic reviews supported by concurrent meta-analyses have confirmed these findings, verifying the belief that cerclage could prolong gestation and improve outcomes among selected twin populations [3,4]. Additionally, an up-to-date retrospective cohort study demonstrated that emergency cerclage, in contrast to ultrasound-guided cerclage, significantly increased gestational length with favorable outcomes among twin pregnancies complicated by cervical insufficiency [5], highlighting the importance of patient-specific clinical context in intervention choice.

Cervical pessary represents a less invasive procedure, with initial studies indicating potential advantages in twin gestations complicated by a short cervix. In the PECEP-Twins study, the insertion of pessary in women with twin pregnancies and cervical length ≤ 25 mm was found to significantly decrease the rate of spontaneous preterm birth (PTB) prior to 34 weeks when compared with expectant management (16.2% vs. 39.4%; RR 0.41) [6]. Likewise, Merced et al. demonstrated that in women with a history of threatened preterm labor and a persistently short cervix, the use of pessary was associated with a decreased rate of PTB prior to 34 weeks (16.4% vs. 32.3%; RR 0.51), less recurrence of threatened preterm labor, and fewer neonates with a birth weight < 2500 g [7]. In contrast to these results, a large, multicenter randomized study comparing pessary and cerclage (with or without progesterone) reported no difference in the rate of PTB prior to 34 weeks. Importantly, however, cerclage was found to have significantly lower rates of PTB prior to 28 weeks and decreased perinatal mortality [8]. However, these findings suggest benefit primarily within narrowly defined clinical subgroups.

Due to conflicting results, meta-analyses were conducted to further evaluate the literature findings. A network meta-analysis reported modest benefits of pessary and cerclage in twins at risk, though it highlighted significant uncertainty across interventions [9]. Moreover, a comprehensive systematic review and meta-analysis further confirmed that cerclage, but not pessary, was linked to improved gestational age and reduced PTB in twin pregnancies with a short cervix or cervical dilation [4], underscoring the need for individualized intervention selection rather than uniform application.

Despite these advances, direct comparative effectiveness evidence between pessary placement, cervical cerclage, and expectant management in twin pregnancies remains limited, largely due to heterogeneity in study design, inclusion criteria, and intervention timing. Differences in research design (e.g., prospective cohort or case series), inclusion criteria (dilation versus short cervix versus post–preterm labor), and intervention timing confound the possibility of direct comparison. Additionally, even though cervical length has been consistently established as a predictor of preterm delivery in twin gestations [10], the predictive value of other aspects of cervical configuration, e.g., diameter or funneling, as modifiers of treatment response and individualized risk, is poorly understood. This study aims to address these knowledge gaps by examining the association between intervention type (pessary, cerclage, or expectant management) and gestational age at delivery in twin pregnancies, within a personalized medicine framework. Additionally, this study determines the prognostic value of cervical features such as length, diameter, and funneling, through descriptive statistics, survival analysis, and multivariable Cox regression methods.

In singleton pregnancies, strong evidence supports the use of vaginal progesterone and cervical cerclage to prevent preterm birth, particularly among women with a prior spontaneous preterm delivery or a short cervical length. Meta-analyses have shown that vaginal progesterone significantly reduces the risk of preterm birth and neonatal morbidity in singleton gestations with a short cervix, and both progesterone and cerclage are effective strategies in high-risk singleton pregnancies [11]. Cervical pessary may also reduce the risk of preterm delivery in singleton pregnancies, though evidence remains uncertain [12].

However, the extrapolation of these interventions to twin pregnancies has yielded inconsistent results. In unselected twin populations, progesterone, cerclage, and pessary have not consistently demonstrated reductions in preterm birth or perinatal morbidity, though subgroup analyses suggest potential benefit in select cases, for instance, in cases with a very short cervix [13].

Preterm birth in twin gestations is multifactorial and may involve infection- or inflammation-driven pathways as well as mechanically mediated cervical factors. Cervical support interventions, such as cerclage or pessary placement, are primarily intended to address mechanical cervical insufficiency and require careful patient selection. This discrepancy highlights the need for a population-specific evaluation of cervical interventions in twin gestations and provides the rationale for the present comparative effectiveness analysis within a carefully selected clinical cohort.

## 2. Methods

### 2.1. Study Design and Population

This retrospective cohort study evaluated the association between cervical characteristics, cervical management strategies, and gestational age at delivery in twin pregnancies. A total of 120 twin pregnancies managed between January 2019 and January 2024 were included in the analysis. The clinical protocols for cervical assessment and management of twin pregnancies at our center remained stable throughout this study.

All women participating in this study were recruited from the University Hospital of Alexandroupolis, a tertiary referral center. In addition, selected patient data from a gynecological referral center in Aschaffenburg, Germany, were used exclusively for comparative control purposes, following approval from the director of the center. The German dataset included 30 twin pregnancies that met identical eligibility criteria and were managed according to comparable clinical protocols. These cases were not analyzed as a separate cohort but were incorporated as controls to enhance the comparative interpretation of cervical management strategies.

The choice of cervical management strategy was based on gestational age, cervical morphology (including cervical length, diameter, and funneling), obstetric history, and overall clinical judgment, reflecting routine tertiary-care practice.

The study protocol was approved by the Institutional Ethics Committee of the University Hospital of Alexandroupolis on 15 October 2021 (protocol RF Number 43760). The study cohort comprised 120 twin pregnancies managed between January 2019 and January 2024. The primary analyzed dataset derives predominantly from the post-approval period (15 October 2021 to January 2024). A limited number of pre-approval cases (*n* = 30, January 2019 to 14 October 2021) were included only for completeness, after full anonymization and consistent with the approved protocol for retrospective data use. This study was conducted within the framework of an institutionally approved PhD project at Democritus University of Thrace, under institutional oversight.

### 2.2. Inclusion and Exclusion Criteria

Inclusion criteria were:

1. Confirmed twin pregnancy.
2. Availability of cervical assessment data, including cervical length, cervical diameter, and/or presence of funneling.
3. Documented cervical management strategy, classified as pessary placement, cervical cerclage, or expectant management.
4. Documented gestational age at delivery.
5. Negative vaginal and cervical microbiological cultures during pregnancy.
6. Normal cervical cytology (Pap smear).

Exclusion criteria included:

1. Positive microbiological cultures suggesting ascending infection.
2. Abnormal Pap smear results.
3. Major fetal structural or chromosomal abnormalities.
4. Twin-to-twin transfusion syndrome requiring fetal intervention.
5. Incomplete clinical or outcome data.

Cervical ultrasound assessment was performed during the second trimester (18–24 weeks of gestation), in accordance with standard clinical practice, and only the first documented cervicometry measurement obtained during pregnancy was included for each patient to ensure the homogeneity of cervical assessment. Cervical diameter was measured transvaginally at the level of the internal cervical os during standard ultrasound cervicometry.

Before any cervical intervention (pessary or cerclage), patients underwent routine clinical and laboratory assessment to exclude active infection, including body temperature measurement, inflammatory markers (white blood cell count and C-reactive protein), and microbiological cultures according to institutional protocols. Cervical interventions were applied in asymptomatic women without clinical evidence of membrane rupture or active infection at the time of assessment. Prophylactic antibiotic therapy was routinely administered following cervical intervention as part of standard care.

### 2.3. Etiologic Focus

By excluding women with positive vaginal or cervical microbiological cultures and abnormal cervical cytology, the study population represents a cohort without microbiological evidence of ascending infection. However, a systematic assessment of inflammatory biomarkers and standardized evaluation of clinical signs of inflammation were not uniformly available.

Consequently, while overt infectious causes of preterm birth were minimized through microbiological and cytological screening, subclinical inflammatory pathways cannot be entirely excluded. Within this context, the analysis focuses on pregnancies in which mechanical cervical dysfunction was considered a relevant clinical factor guiding management, based on cervical morphology, gestational age, obstetric history, and overall clinical assessment.

### 2.4. Data Collection and Variables

Collected variables included gestational age at delivery, cervical length (≤2.4 cm vs. >2.4 cm), cervical diameter (≤10 mm vs. >10 mm), presence or absence of funneling, and cervical management strategy (pessary, cerclage, or no intervention). Preterm birth was defined as delivery before 33 completed weeks of gestation. This threshold was selected to focus on earlier, clinically more severe prematurity, which is associated with substantially higher neonatal morbidity and mortality compared with later preterm birth.

Information on the mode of conception and parity was available for all participants. Of the 120 women included, 68 (56.7%) conceived via in vitro fertilization (IVF), and all IVF pregnancies occurred in primiparous women. Among the remaining 52 non-IVF pregnancies (43.3%), 10 women (19.2%) were multiparous (para 2), while the majority (42/52; 80.8%) were also primiparous. Overall, 110 of 120 women (91.7%) were primiparous. All participants received vaginal progesterone throughout the study period, in accordance with our standard clinical practice for twin pregnancies at increased risk of preterm birth.

Cervical interventions were selected based on obstetric history, gestational age, and cervical findings, according to routine clinical practice. Cerclage placement was performed as either history-indicated (prophylactic) in women with prior cervical insufficiency or prior second-trimester loss/preterm birth, typically before 23 weeks’ gestation, or ultrasound-indicated (therapeutic) in cases of significant cervical shortening identified during follow-up, at the discretion of the treating team. Pessary insertion was offered as a less invasive cervical support option in selected cases based on cervical assessment and overall clinical context.

Variables such as smoking status, socioeconomic characteristics, inflammatory biomarkers (e.g., C-reactive protein, white blood cell count), and detailed obstetric history were not uniformly available across the entire retrospective study period and were therefore not included in multivariable analysis. Information on chorionicity was not uniformly documented across the retrospective study period and therefore could not be included in the analysis. To avoid bias related to incomplete data and inconsistent documentation, only variables systematically recorded for all participants were analyzed. This study was not designed to model overall preterm birth risk but rather to compare gestational outcomes across cervical management strategies within this selected clinical context.

### 2.5. Statistical Analysis

Statistical analyses were conducted using IBM SPSS Statistics for Windows, Version 22.0 (IBM Corp., Armonk, NY, USA). Descriptive statistics were used to summarize maternal and cervical characteristics, stratified by intervention type and preterm birth status.

Categorical variables were compared using the chi-square test or Fisher’s exact test, as appropriate. Gestational age distributions across intervention groups were visualized using kernel density plots and boxplots.

Time-to-event analysis was performed using Kaplan–Meier survival curves, with delivery as the event of interest. Survival curves were compared across intervention groups using the log-rank (Mantel–Cox) test. Multivariable Cox proportional hazards regression analysis was used to estimate adjusted hazard ratios (HRs) and 95% confidence intervals (CIs) for earlier delivery. Covariates included intervention type (with cerclage as the reference category), cervical length, cervical diameter, and funneling. A two-tailed *p*-value < 0.05 was considered statistically significant.

## 3. Results

### 3.1. Statistical Analysis of Twin Pregnancies: Gestational Age at Delivery by Intervention Type

To investigate the relationship between the type of intervention and gestational age at delivery in twin pregnancies, two visualizations were generated: a kernel density plot and a boxplot. The dataset included clinical characteristics of twin pregnancies and recorded whether women received a cerclage, a pessary, or expectant management.

**Figure 1:**
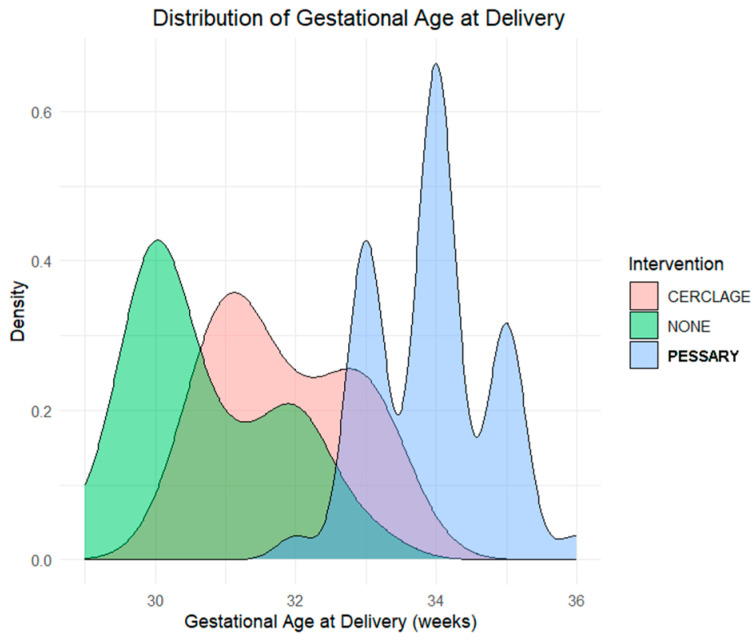
Gestational age at delivery by intervention type in twin pregnancies. Kernel density plot of gestational age shows that pessary group delivered later on average.

**Figure 2:**
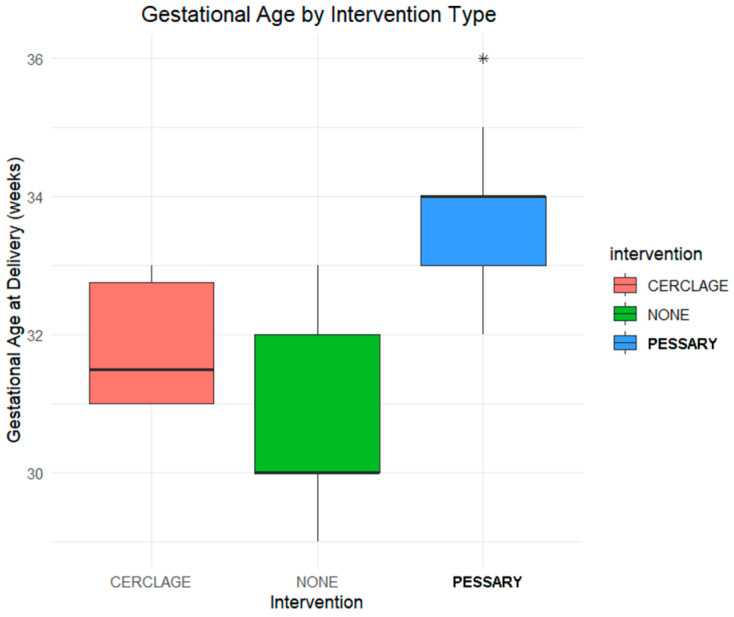
Boxplot comparing gestational age across intervention types. Pessary group had highest median and least variability.

The kernel density plot (Figure 1) illustrates the distribution of gestational age at delivery in our population of 120 twin pregnancies by intervention type. A clear rightward shift is observed in the pessary group, indicating that these patients tend to deliver at later gestational weeks. The highest peak of density lies around 34 weeks for pessary-treated patients, compared to approximately 31–32 weeks for the other two groups. The boxplot (Figure 2) confirms this pattern: the pessary group shows the highest median gestational age and the least variability. Conversely, the no-intervention group exhibits the lowest median and broader spread, suggesting a greater risk of earlier delivery. The cerclage group also displays a relatively earlier delivery distribution compared to the pessary group.

These visualizations demonstrate differences in the distribution of gestational age at delivery across intervention groups, with later gestational ages observed in the pessary group compared with the cerclage and no-intervention groups.

### 3.2. Descriptive Statistics in Twin Pregnancies Stratified by Preterm Birth (<33 Weeks)

To explore the association between cervical characteristics, intervention types, and the risk of preterm birth in twin pregnancies, a descriptive analysis was performed (Table 1). The dataset was stratified based on whether delivery occurred before 33 weeks of gestation (preterm = 1) or not (preterm = 0). Five categorical variables were analyzed: cervical length group (≤2.4 cm vs. >2.4 cm), cervical diameter group (≤10 mm vs. >10 mm), presence of funneling, pessary use, and cerclage use. The results are presented as counts and percentages per group, along with *p*-values derived from chi-square or Fisher’s exact tests, depending on expected frequencies.

In unadjusted comparisons (Table 1), the cervical diameter group, funneling status, and pessary use differed between pregnancies delivering before versus after 33 weeks (all *p* < 0.001), whereas cervical length group (*p* = 0.161) and cerclage use (*p* = 0.111) did not.

### 3.3. Kaplan–Meier Survival Analysis in Twin Pregnancies: Gestational Age by Intervention

**Figure 3:**
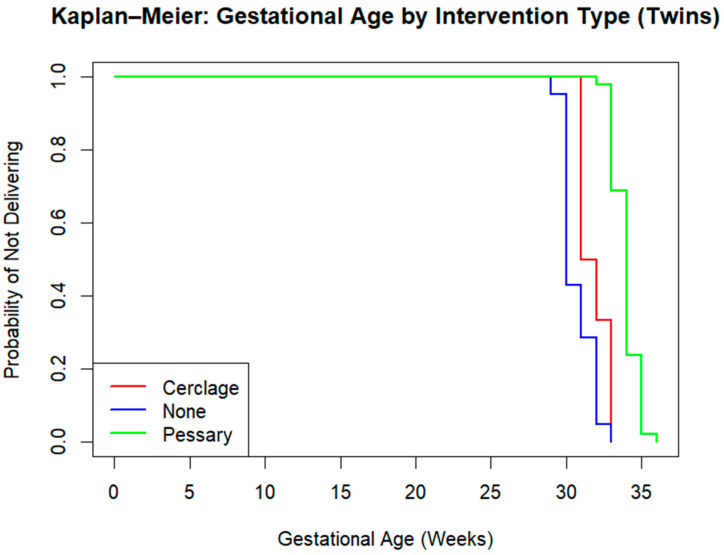
Kaplan–Meier survival curves for gestational age at delivery in twin pregnancies.

To assess the impact of different intervention strategies (pessary, cerclage, or no intervention) on the prolongation of pregnancy in twin gestations, we performed a Kaplan–Meier survival analysis. The time-to-event variable was gestational age at delivery (week), and all events were coded as delivery (i.e., event = 1 for all observations). The analysis evaluates the “survival” of the pregnancy over time, that is, the probability of not delivering as gestational weeks progress. Below we present the Kaplan–Meier survival plot (Figure 3) along with the statistical results from the log-rank (Mantel–Cox) test (Table 2). The survival curves clearly separate across the three intervention types (Figure 3):

- Pessary group (green line).
- Cerclage group (red line).
- No-intervention group (blue line).

Kaplan–Meier curves for gestational age at delivery differed by intervention group (Figure 3). The pessary group showed later deliveries overall compared with the cerclage and no-intervention groups.

The log-rank test revealed a highly statistically significant difference between the three intervention groups (*p* < 2 × 10^−16^) (Table 2).

- The no-intervention group had far more preterm deliveries than expected (Observed = 21 vs. Expected = 3.31), contributing the most to the chi-square statistic.
- The cerclage group also delivered earlier than expected but had a smaller sample size.
- The pessary group, on the other hand, showed fewer observed events than expected (Observed = 93 vs. Expected = 114.87), indicating an association with prolonged gestational duration.

### 3.4. Multivariable Cox Proportional Hazards Model in Twin Pregnancies

To further investigate which factors independently influence the timing of delivery in twin pregnancies, we conducted a multivariable Cox proportional hazards regression. The outcome variable was gestational age at delivery (in weeks), treated as a time-to-event variable, with the assumption that all pregnancies reached the event of delivery (event = 1). The analysis aimed to explore the association of four key predictors with the hazard of earlier delivery: the type of intervention used (pessary, cerclage, or none), grouped cervical length (≤2.4 cm vs. >2.4 cm), grouped cervical diameter (≤10 mm vs. >10 mm), and the presence of funneling. This multivariate model allows us to estimate adjusted hazard ratios (HRs) for each factor while controlling for the others.

The results indicate that the use of a pessary is significantly associated with a reduced risk of early delivery. Specifically, women in the pessary group had a hazard ratio (HR) of 0.088, with a 95% confidence interval ranging from 0.035 to 0.220. This suggests they had an approximately 91% lower risk of earlier delivery compared to the reference group (cerclage), and the association was highly statistically significant (*p* < 0.001). Pessary use was associated with a lower hazard of earlier delivery in this cohort.

On the other hand, women who received no intervention (neither cerclage nor pessary) had an increased hazard of delivery (HR = 2.44), with a confidence interval between 0.94 and 6.33. Although this result was not statistically significant at the 0.05 level (*p* = 0.067), it suggests a trend indicating that the absence of any preventive intervention may be associated with a higher likelihood of earlier delivery. This association did not reach statistical significance (*p* = 0.067)

Cervical length ≤ 2.4 cm (HR = 1.21, *p* = 0.548) and cervical diameter ≤ 10 mm (HR = 0.90, *p* = 0.823) were not statistically significant predictors in the adjusted model. Funneling (Yes) was associated with HR = 0.35 (95% CI: 0.16–0.78, *p* = 0.010) (Table 3).

## 4. Discussion

This retrospective observational study on twin pregnancies demonstrated that pessary use was associated with prolonged gestational duration and a lower hazard of prematurity compared with cerclage or expectant management. The association was supported by descriptive statistics, Kaplan–Meier survival curve analysis, and multivariable Cox regression modeling, showing a 91% reduction in the hazard of early delivery (HR = 0.088, 95% CI: 0.035–0.220, *p* < 0.001). Given the retrospective design, these findings reflect associations rather than causality. These findings support an individualized approach to preterm birth prevention, in which intervention choice is guided by patient-specific risk profiles rather than a uniform strategy, and they highlight the possible benefits associated with the use of a pessary for carefully selected high-risk twin pregnancies.

Consistent with the distributional patterns observed in the kernel density plot and boxplot, the use of pessaries may be associated with prolonged pregnancy duration compared to cerclage or expectant management, though this observation is descriptive and requires confirmation through formal statistical analyses.

### 4.1. Comparison to Existing Evidence

The role of cervical pessaries in twin pregnancies is controversial. Large, randomized trials like the ProTWIN study and several subsequent meta-analyses have shown modest benefits from the use of pessaries in cases of unselected twin pregnancies [13]. However, subgroup analyses and targeted studies suggest possible benefits for women with a shortened cervix. In a randomized trial, Merced et al. [6] presented evidence that the use of pessary in women with twin pregnancies at risk of preterm delivery significantly reduced the likelihood of delivery before 34 weeks. Our results support this evidence, reinforcing the concept that therapeutic efficacy may depend on individualized patient selection and underlying cervical risk, emphasizing the importance of the careful selection of the patient in order to maximize the effect of pessary therapy.

By contrast, cerclage efficacy in twin gestations is less consistently supported. Although some studies suggest a possible decrease in early preterm delivery (<28 weeks) and perinatal mortality [14], other studies have failed to demonstrate a clear benefit consistently, particularly in situations where cervical shortening is the only risk criterion examined [15]. In our cohort, cerclage use was associated with shorter gestational duration compared with pessary use and did not significantly reduce the hazard of early delivery. These findings should be interpreted cautiously, given the small number of cerclage cases and potential selection bias. Nevertheless, they underscore the limitations of extrapolating interventions developed for singleton pregnancies to twin gestations without individualized risk assessment.

A recent systematic review by Saccone et al. [16] found that for twin pregnancies involving a short cervix, the use of a pessary reduced preterm birth before 34 weeks by approximately 30% when compared to expectant management, but cerclage failed to show comparable benefits. These results support the proposition that pessary use may offer superior efficacy within this defined population, perhaps through its capability to mechanistically shift the cervical load and modify the uterocervical angle. Our findings extend this literature by suggesting that, when cervical features and intervention type are considered together, pessary placement may be associated with more favorable gestational outcomes within selected twin populations. However, this observation should be considered hypothesis-generating rather than definitive.

By comparison, the PECEP-Twins trial [5], in which women with short cervices and twin pregnancies were randomized to pessary intervention or expectant management, found a non-significant trend towards lower rates of preterm births in the pessary group. This result implies that variables including study design, participant compliance, and population risk profile may have an impact on effect size. The variations we see between trials underscore the heterogeneity within twin pregnancy cohorts and highlight the importance of personalized medicine approaches that identify subgroups most likely to benefit from intervention. The results of our study, based on a clinical cohort that had a high proportion of women with short cervices, probably reflect this particular protective effect.

Seetho et al. [17] reported that 65.1% of twin pregnancies resulted in preterm birth, specifically in monochorionic twins (AOR 2.06, 95% CI 1.29–3.30) and antepartum complications. This highlights the requirement for prophylactic intervention based on chorionicity to avoid preterm delivery in high-risk twins. Our case series showed an association between pessary use and prolonged gestation within this selected cohort, independently of individual cervical measurement thresholds. This finding supports a risk-stratified model of care, in which chorionicity and clinical complexity are integrated into individualized prevention strategies. Pessary use was associated with more favorable gestational outcomes in selected high-risk twin pregnancies, particularly those with increased clinical complexity, which is consistent with Seetho et al.’s risk stratification. These results support the rationale for prophylactic interventions based on individual risk and chorionicity, rather than applying a uniform approach.

In the comparative analysis by Tsikouras et al. (2018) [18], the authors noted that the efficacy of both Arabin pessary and cerclage appeared to be lower in multiple gestations compared to singleton pregnancies, reflecting the higher baseline risk of preterm birth in twins. Additionally, they reported that although use of a pessary may be associated with some benefits in selected twin pregnancies, the evidence to date is not sufficient to support its use as a preventive method on a large scale, thus highlighting the need for completed randomized trials in this group [18]. Our findings contribute to this evolving evidence base by identifying clinical contexts in which pessary use may be the most effective, thereby informing future trial design.

Tanaka et al. [19] demonstrated that both cervical shortening and elevated inflammatory biomarkers are predictors of spontaneous preterm birth in asymptomatic twin pregnancies. In the present study, cervical length did not retain statistical significance in multivariable analysis, possibly reflecting the modifying effect of cervical interventions. This suggests that cervical measurements may be more useful for guiding individualized management decisions than for serving as independent prognostic markers once intervention type is considered.

In our cohort, unadjusted stratification by delivery < 33 weeks showed significant between-group differences in several cervical and management variables (Table 1). Pregnancies delivering after 33 weeks more frequently had a cervical diameter > 10 mm and were managed with a pessary, whereas the preterm group more often had a cervical diameter ≤ 10 mm and did not receive a pessary. In contrast, cervical length did not differ significantly between groups, and cerclage use showed no statistically significant difference in these unadjusted comparisons. These patterns should be interpreted cautiously, as they may reflect clinical selection and treatment allocation in routine practice rather than isolated prognostic effects of individual cervical parameters.

Unexpectedly, cervical funneling was associated with a reduced hazard of early delivery. This finding contrasts with most of the existing literature, which associates funneling with increased preterm birth risk [20]. This paradoxical result should be interpreted cautiously and may reflect selection bias, differential clinical surveillance, or unmeasured co-interventions rather than a true protective effect.

### 4.2. Clinical Implications

In this cohort of twin pregnancies, pessary use was associated with prolonged gestation and a markedly reduced risk of preterm birth compared with cerclage or no intervention, whereas cerclage did not demonstrate an independent protective effect. These findings support a personalized, risk-adapted approach to intervention selection in twin pregnancies. Clinical decision-making should remain individualized, taking into account existing evidence that supports cerclage in cases of advanced cervical insufficiency, while prospective randomized trials are needed to clarify the relative role of both interventions.

### 4.3. Strengths and Limitations

An important strength of this study is the use of multiple complementary analytical approaches, including time-to-event modeling, which enhances clinical interpretability. The uniform administration of vaginal progesterone to all participants may be considered a strength, as it reduced treatment-related heterogeneity across intervention groups. However, the retrospective design, unequal group sizes, and limited availability of certain confounding variables restrict causal inference. Additionally, although pregnancies with microbiological evidence of infection were excluded, subclinical inflammatory processes cannot be entirely ruled out. Unexpected findings related to funneling require cautious interpretation and further investigation in prospective studies. This counterintuitive direction may reflect selection effects, measurement or coding variability, or unrecorded co-interventions in routine care rather than a biologically protective effect.

Although the mode of conception and parity were documented, these variables were not included in multivariable modeling due to the predominance of primiparity and the high proportion of IVF pregnancies, which limited meaningful stratified analysis. Chorionicity and zygosity were not uniformly documented across the retrospective study period and therefore could not be included in the analysis.

## 5. Conclusions

This study examined the association between cervical characteristics and the risk of preterm delivery (<33 weeks) in twin pregnancies, focusing on the comparative effectiveness of pessary placement, cervical cerclage, and expectant management. A combination of descriptive statistics, Kaplan–Meier survival analysis, and multivariable Cox proportional hazards modeling was applied to identify clinically relevant associations with early delivery in a selected high-risk twin population.

Pessary use in twin pregnancies was consistently associated with a lower hazard of early delivery. In contrast, cervical cerclage did not demonstrate an independent protective association, and the absence of any intervention was associated with a higher risk of early delivery. These findings emphasize the importance of individualized treatment selection rather than reliance on cervical anatomical parameters alone.

Given the retrospective study design and the potential for residual confounding, these findings should be interpreted as associative rather than causal. Future studies should aim to validate these findings in larger, prospectively recruited cohorts, ideally incorporating randomized allocation of interventions. The integration of dynamic cervical measurements, biomarker profiles, and detailed obstetric history may further enhance predictive accuracy. Ultimately, a personalized medicine framework integrating clinical, anatomical, and contextual factors may help optimize preterm birth prevention strategies in twin pregnancies.

## Figures and Tables

**Table 1:** Descriptive statistics.

	0 (Non-Preterm)	1 (Preterm)	*p*-Value
N	94	26	
Cervical Length			0.161
>2.4 cm	7 (7.4%)	5 (19.2%)	
≤2.4 cm	87 (92.6%)	21 (80.8%)	
Cervical Diameter			<0.001
>10 mm	91 (96.8%)	12 (46.2%)	
≤10 mm	3 (3.2%)	14 (53.8%)	
Funneling			<0.001
NO	5 (5.3%)	15 (57.7%)	
YES	89 (94.7%)	11 (42.3%)	
Pessary Use			<0.001
NO	3 (3.2%)	24 (92.3%)	
YES	91 (96.8%)	2 (7.7%)	
Cerclage Use			0.111
NO	62 (66.0%)	22 (84.6%)	
YES	32 (34.0%)	4 (15.4%)	

**Table 2:** Log-rank test results.

Intervention Group	N	Observed Events	Expected Events	(O–E)^2^/E	(O–E)^2^/V
Cerclage	6	6	1.81	9.68	11.9
No intervention	21	21	3.31	94.41	119.5
Pessary	93	93	114.87	4.17	125.5

**Table 3:** Regression output table.

Predictor	Hazard Ratio (HR)	95% CI (Lower–Upper)	*p*-Value	Significance
No Intervention	2.44	0.94–6.33	0.067	marginal
Pessary	0.088	0.035–0.220	<0.001	***
Cervical Length ≤ 2.4	1.21	0.65–2.22	0.548	n.s.
Diameter ≤ 10 mm	0.90	0.37–2.20	0.823	n.s.
Funneling (Yes)	0.35	0.16–0.78	0.010	**

*** means high significance; n.s. no significance; ** intermediate significance.

## Data Availability

The raw data supporting the conclusions of this article will be made available by the authors on request.
